# Expression of Hexokinase in Stomata of Citrus Fruit Reduces Fruit Transpiration and Affects Seed Development

**DOI:** 10.3389/fpls.2020.00255

**Published:** 2020-03-06

**Authors:** Nitsan Lugassi, Gilor Kelly, Tal Arad, Chagai Farkash, Yossi Yaniv, Yelena Yeselson, Arthur A. Schaffer, Eran Raveh, David Granot, Nir Carmi

**Affiliations:** ^1^Institute of Plant Sciences, Agricultural Research Organization, The Volcani Center, Bet Dagan, Israel; ^2^Department of Fruit Tree Sciences, Institute of Plant Sciences, Agricultural Research Organization, Gilat Research Center, Negev, Israel

**Keywords:** sugar-sensing, hexokinase, fruit stomata, fruit transpiration, floral organs

## Abstract

The temporal formation and spatial distribution of stomata on the surface of citrus floral organs and, specifically, on the ovule from which the fruit develops, were analyzed using citrus plants that express green fluorescent protein (GFP) under the guard cell-specific *KST1* promoter. Stomata are found on the style, sepal, and anther of the closed flower and on ovules from the stage of anthesis. It has previously been shown that hexokinase (HXK) mediates sugar-sensing in leaf guard cells and stimulates stomatal closure. The activity and response of citrus fruit stomata to sugar-sensing by HXK was examined using plants that express HXK under the *KST1* promoter. Those plants are referred to as GCHXK plants. The transpiration of young green GCHXK citrus fruits was significantly reduced, indicating that their stomata respond to sugar similar to leaf stomata. Toward fruit maturation, fruit stomata are plugged and stop functioning, which explains why WT and GCHXK mature yellow fruits exhibited similar water loss. Seeds of the GCHXK plants were smaller and germinated more slowly than the WT seeds. We suggest that the stomata of young green citrus fruits, but not mature yellow fruits, respond to sugar levels via HXK and that fruit stomata are important for proper seed development.

## Introduction

Stomata are the gates that allow the exchange of gases between the atmosphere and the inner tissues of the plant. During the process of gas exchange, the plant transpires water vapor into the surrounding atmosphere and CO_2_ for photosynthesis enters the plant. The spatial and temporal distribution of stomata on leaves has been studied extensively over the past decades (Geisler and Sack, 2002; Nadeau and Sack, 2002; Kelly et al., 2017). In Arabidopsis, stomata appear on the cotyledons immediately after germination and, soon after, on the edges of true leaves, and the densities of the stomata on those plant parts increase in the following days (Kelly et al., 2017). Stomata are also found on the hypocotyl and stem and have been observed on Arabidopsis and *Lilium* hyb. *enchantment* anthers and the fruit skins of apple (*Malus domestica*), banana (*Musa acuminata*), passionfruit (*Passiflora edulis*), pitaya (*Hylocereus megalanthus*), and citrus (*Citrus unshiu*) (Johnson and Brun, 1966; Blanke and Lenz, 1989; Clement et al., 1997; Wilson et al., 2011; Sánchez et al., 2013; Hiratsuka et al., 2015; Wei et al., 2018). Yet, the presence and temporal development of stomata on other flower parts, such as the petal, style, and ovule, have been the subject of very little study (Huang et al., 2018).

In most plants, leaf stomata open in response to light, to allow the entry of atmospheric CO_2_ for photosynthesis, and this happens at the expense of extensive water loss via the open stomata. At night, the stomata close to prevent water loss. It has been shown that leaf stomata close in response to increasing sugar levels and that, within guard cells, this process is mediated by HXK (Kelly et al., 2013; Lugassi et al., 2015; Kottapalli et al., 2018). HXK is the only enzyme in plants that can phosphorylate glucose and may also phosphorylate fructose (Granot, 2007, 2008). In addition to its catalytic phosphorylation activity, HXK also functions as a sugar sensor independent of its phosphorylation activity (Moore et al., 2003). It is assumed that HXK senses the level of glucose and fructose and generates a signal that activates the ABA pathway that closes stomata (Kelly et al., 2013). Stomatal closure by sugars and HXK is a conserved trait that allows for coordination between photosynthesis and transpiration (Kelly et al., 2013; Lugassi et al., 2015; Kottapalli et al., 2018; Granot and Kelly, 2019). It has been shown in some fruits, such as purple passionfruit, yellow pitaya, banana, and apple, that fruit stomata respond in a similar way to leaf stomata, opening in response to light, high temperatures, and high humidity (Johnson and Brun, 1966; Blanke and Lenz, 1989; Sánchez et al., 2013). Yet, whether fruit stomata also respond to sugar is not known. We used citrus plants expressing either GFP or HXK under the guard cell-specific promoter *KST1* to follow the development of stomata on the various organs of citrus flowers and to examine the response of the stomata of citrus fruits to sugar.

## Materials and Methods

### Plant Material and Growth Conditions

Experiments were conducted on *Troyer citrange* (*Citrus sinensis* “Washington” sweet orange × *Poncirus trifoliata*). *T. citrange* explants were transformed with *KSTpro:GFP* and *KSTpro:HXK1* constructs that express *GFP* or *HXK1* under the *KST1* promoter, respectively, as described previously by Lugassi et al. (2015). Plants transformed with *KSTpro:GFP* or *KSTpro:HXK1* are referred to as GCGFP and GCHXK (standing for guard-cell GFP and guard-cell HXK, respectively). Plants were grown in 10-L pots that contained (w/w) 30% vermiculite, 30% peat, 20% tuff, and 20% perlite (Even Ari, Israel). WT (untransformed), GCGFP, and GCHXK plants were vegetatively propagated by the grafting of shoots onto *T. citrange* rootstocks. Two independent lines of GCHXK plants, GCHXK1 and GCHXK5, were used in some of the experiments, based on the availability of the necessary plant material. WT, GCHXK1, and GCHXK5 plants were of the same age, were grafted at the same time, and were trimmed periodically to be similar in size. The grafted plants were grown in a temperature-controlled greenhouse (25–30°C in the summer and 15–25°C in the winter) under natural light conditions.

### Confocal Microscopy Imaging

Images were acquired using the Olympus IX 81 inverted laser scanning confocal microscope (Fluoview 500; Olympus Corporation, Tokyo, Japan) equipped with a 488-nm argon ion laser and a 60 × 1.0 numerical aperture PlanApo water immersion objective (Olympus). Green fluorescent protein was excited by 488-nm light and the emission was collected using a BA 505–525 filter. A BA 660 IF emission filter was used to observe chlorophyll autofluorescence. Confocal optical sections were obtained in 0.5- to l-μm increments. The images were color-coded green for GFP and magenta for chlorophyll autofluorescence.

The image presented in Figure 7 was made using a Leica SP8 laser-scanning microscope (Leica, Wetzlar, Germany) equipped with a solid-state laser with 488 nm light, HC PL APO CS 63×/1.2 water immersion objective (Leica, Wetzlar, Germany) and Leica Application Suite X software (LASX, Leica, Wetzlar, Germany). Images of GFP signal were acquired using the 488-nm laser line and emission was detected with a HyD (hybrid) detector in a range of 500–525 nm. For reflection microscopy, a 488-nm laser was used and light reflected into a band between 480 and 495 nm.

### Distribution of Stomata on Various Parts of Citrus Fruits and Flowers

To evaluate the temporal formation and spatial distribution of stomata on various parts of citrus flowers, we collected five closed flowers and five open flowers and analyzed the distribution of stomata on the various floral organs. The same analyses were repeated in two sequential flowering seasons, with the same number of flowers examined each season.

Analyses of the stomata on fruit surfaces were conducted twice, with similar results. In the first analysis, a comparison was made between at least five stomata from each of three ripe fruits and three green fruits. In the second analysis, which was conducted the following season (the results of which are presented in Figure 7), the comparison was made between at least five stomata taken from each green or ripe parts of three breaker fruits (total of 10 stomata per fruit).

### Measurement of the Transpiration of Green Fruits

Fruit transpiration rates were measured on intact 4-cm-diam. green fruits from plants grown in a greenhouse using the LI-1600 steady-state porometer (LI-COR, Lincoln, NE, United States). Measurements were conducted between 9:00 a.m. and 10:00 a.m. on all of the available fruits: eight WT fruits and 12 GCHXK1 fruits. The ambient light intensity was 550 μmol m^–2^ s^–1^, the temperature was 23°C, and the relative humidity was 55%.

### Water Loss of Ripe Yellow Fruits

Six ripe yellow fruits of similar weight from GCHXK and WT plants (average weights of 83.8 g for the WT and 82.7 g for GCHXK1) were incubated soon after harvest for 5 days under long-day conditions (16 h light/8 h dark photoperiod) at 25°C and 50% relative humidity. Weight loss during the incubation time was measured and is presented as a percentage from the initial weight (Figure 6).

### Stomatal Measurements

Stomatal aperture and density were determined using the rapid imprinting technique described by Geisler and Sack (2002). The rapid imprinting technique with fast-drying dental resin allowed us to score a large number of stomata from independent biological samples from each experiment. In brief, light-bodied vinyl polysiloxane dental resin (Zhermack, Badia Polesine, Italy) was attached to the fruit surface and then removed as soon as it had dried (1 min). The resin epidermal imprints were covered with nail polish, which was removed once it had dried and served as a mirror image of the resin imprint. The nail-polish imprints transferred to microscope slides and photographed under a bright-field inverted microscope (1M7100; Zeiss, Welwyn Garden City, Hertfordshire, United Kingdom) on which a Hitachi HV-D30 CCD camera (Hitachi, Tokyo, Japan) was mounted. Stomatal images were later analyzed using the IMAGEJ software (Bethesda, MD, United States) fit-ellipse tool to determine aperture size or stomatal density. A microscopic ruler (Olympus, Tokyo, Japan) was used for the size calibration.

The stomatal response to sucrose in WT fruit rind was assayed with rind disks taken from six different green, 4-cm-diam. fruits (one disc from each fruit in each treatment). Rind disks were each 1 cm in diameter and approximately 2 mm thick. The samples were immersed for 2.5 h in either artificial xylem sap (Wilkinson and Davies, 1997) or artificial xylem sap containing 200 mM sorbitol (as an osmotic control), 100 mM sucrose (Suc), and 200 mM Suc. Ambient light intensity was around 500 μmol m^–2^ s^–1^. Epidermal imprints were then taken and stomatal aperture was measured. From each of the six imprints of each treatment, 10 randomly selected stomata were analyzed. Stomatal density was measured using 4-cm-diam., green fruits, with six biological repeats for the WT and seven biological repeats for each GCHXK line. More than 330 stomata were counted for each line in fields of 0.1 mm^2^.

### Seed Germination

The germination rate of GCHXK seeds was examined by sowing 68 seeds of the WT and GCHXK1 plants, and 40 seeds of the GCHXK5 plants. Seeds were collected from fully mature fruits. The seeds were divided into 17 groups of four seeds each for the WT and GCHXK1 (total of 68 seeds for each line) and 10 groups of 4 seeds each for the GCHXK5 line (total of 40 seeds). The proportion of seeds that germinated was calculated based on the average germination of the groups over a period of 28 days.

### Assays of Sugar Levels in Fruit Juice

Juice was collected from three samples of mature yellow fruits of each line. For the WT and GCHXK1, the juice of each sample was collected from two different fruits. For GCHXK5 the juice of each sample was collected from one fruit. Samples were centrifuged at 15,000 rotations/min for 15 min and then filtered through a 0.22-μm nylon syringe. Sucrose, fructose, and glucose contents were determined by HPLC. The HPLC system consisted of a Shimadzu LC10AT solvent delivery system and a Shimadzu RID10A refractive index detector. Separation was carried out on an Alltech 700 CH Carbohydrate Column (Alltech, Deer-Weld, IL, United States) maintained at 90°C with a flow rate of 0.5 ml/min, according to the manufacturer’s recommendations.

### Measurement of Total Soluble Solids

The total soluble solids (TSS) content of the juice of mature yellow fruits was determined with a PAL-1 digital refractometer (Atago, Tokyo, Japan). Each measurement included three samples. For the WT and GCHXK1, the juice of each sample was collected from two different fruits. For GCHXK5, the juice of each sample was collected from one fruit.

## Results

### Temporal Formation and Spatial Distribution of Stomata on Various Parts of Citrus Flowers

**FIGURE 1:**
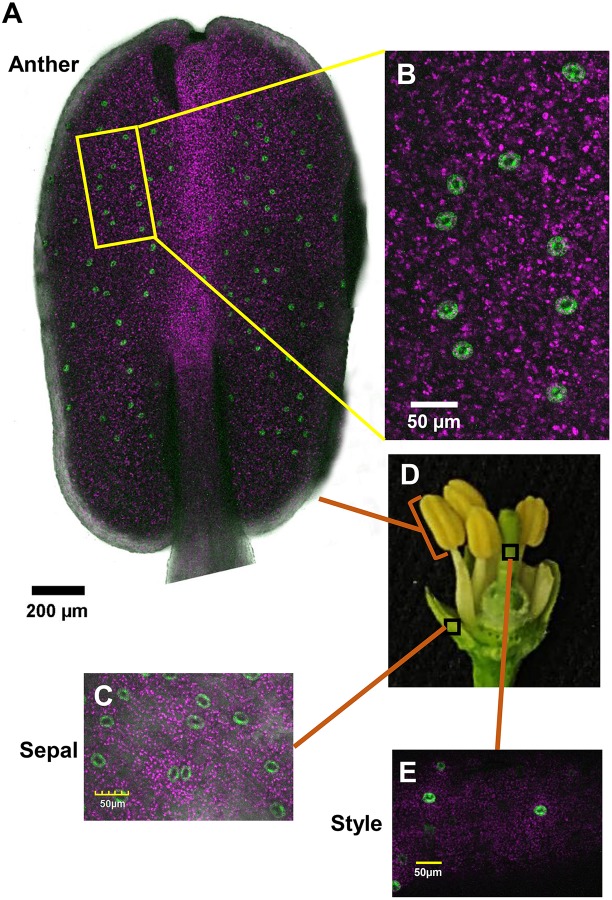
Distribution of stomata on the organs of a closed citrus flower. The confocal images **A–C** and **E** are merged images of white light, chlorophyll autofluorescence (stained magenta), and GFP fluorescence (stained green). Flowers were harvested from GCGFP plants. **(A)** Confocal image of an anther removed from a closed flower. **(B)** Enlargement of the square in part **A**. **(C)** Confocal image of a sepal removed from a closed flower, as indicated in part **D**. **(D)** Dissection of a closed flower revealing the floral organs. **(E)** Confocal image of a style removed from a closed flower. Analyses were performed on five biological replicates.

**FIGURE 2:**
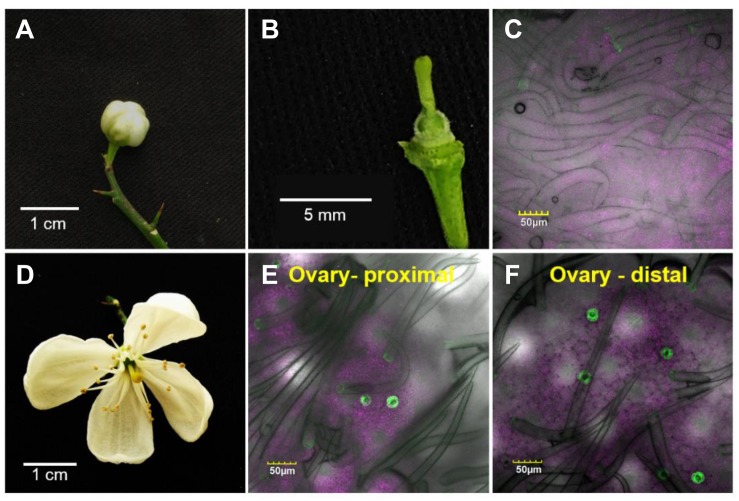
Temporal and spatial distribution of the stomata on a citrus ovary. The confocal images **C**, **E**, and **F** are merged images of white light, chlorophyll autofluorescence (stained magenta), and GFP fluorescence (stained green). Samples were taken from GCGFP plants. The images in the lower row were taken from flowers 3 days older than the images shown in the upper row. **(A)** A closed GCGFP flower. **(B)** Binocular image of a dissected closed flower, revealing the ovary. **(C)** Confocal image of an ovary from a closed flower. **(D)** An open GCGFP flower. **(E)** Confocal image of the proximal part of an ovary from an open flower. **(F)** Confocal image of the distal part of an ovary from an open flower. Analyses were performed on five biological replicates.

**FIGURE 3:**
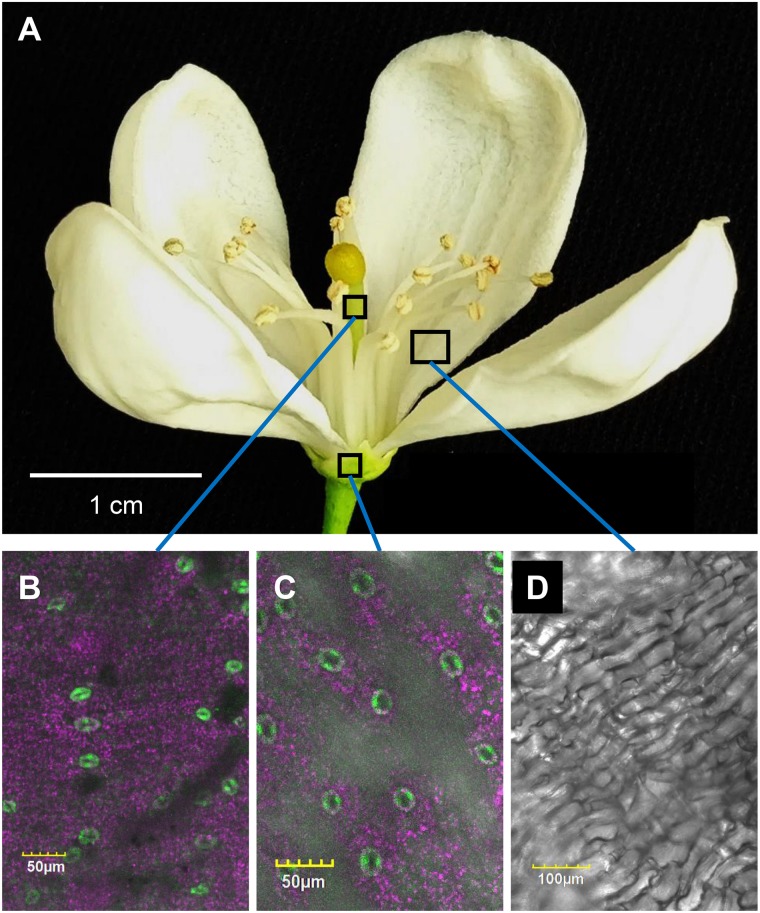
Distribution of stomata on the organs of an open citrus flower. The confocal images **B**, **C**, and **D** are merged images of white light, chlorophyll autofluorescence (stained magenta), and GFP fluorescence (stained green). Flowers were harvested from GCGFP plants. **(A)** An open GCGFP flower. **(B)** Confocal image of a style removed from an open flower, as indicated in panel **A**. **(C)** Confocal image of a sepal removed from an open flower, as indicated in panel **A**. **(D)** Confocal image of a petal removed from an open flower, as indicated in panel **A**. Analyses were performed on five biological replicates.

To monitor the appearance of stomata on citrus flowers and fruits, we used transgenic *T. citrange* (*C. sinensis* “Washington” sweet orange × *P. trifoliata*) containing the *KSTpro:GFP* that drives guard-cell expression of GFP (Lugassi et al., 2015; Kelly et al., 2017). The immediate and constitutive expression of *KSTpro:GFP* in newly formed guard cells (Kelly et al., 2017) allowed us to monitor the appearance of stomata easily and accurately. We started at early stages of flower development, when the flowers were still closed. At this stage, stomata were found on anthers, styles, and sepals (Figure 1), but not on stigmas, filaments, or ovules (which eventually develop into fruits; Figures 2A–C). When the flower opens about 3 days later (Figures 2D, 3A), stomata are seen on the proximal and distal parts of the ovules (Figures 2E,F), but are more abundant on the style and sepal (Figures 3B,C). No stomata were seen on petals, filaments, or stigmas, even at later stages (Figure 3D). We concluded that stomata appear on the styles, anthers, and sepals of flowers that are still closed and on ovules at anthesis.

### The Stomata of Young Citrus Fruits Respond to Sucrose

**FIGURE 4:**
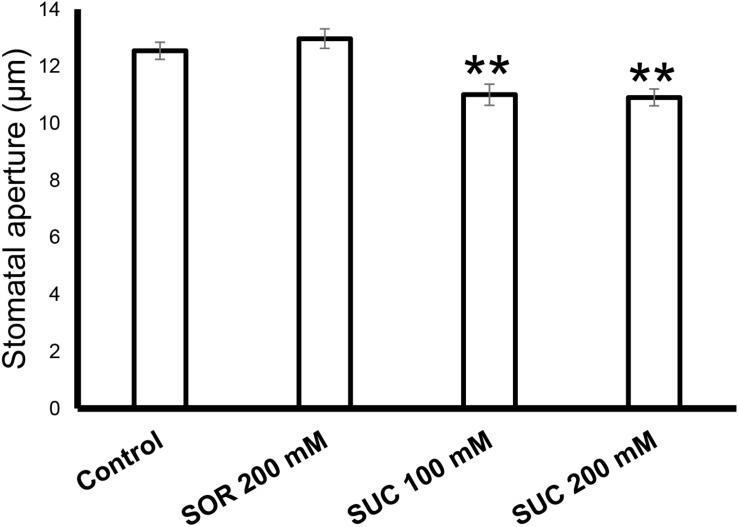
Sugar stimulates the closure of citrus fruit stomata. The stomatal response to sucrose in WT fruit rind was assayed with rind discs (taken from six fruits) that were immersed for 2.5 h in artificial xylem sap (Wilkinson and Davies, 1997), artificial xylem sap containing 200 mM sorbitol (as an osmotic control), 100 mM sucrose (Suc), and 200 mM Suc. Epidermal imprints were then taken and the apertures of 60 stomata from each treatment were measured. Data are displayed as means ± SE. Asterisks denote significant differences relative to the WT (*t*-test, *P* < 0.01).

To check the response of citrus fruits’ stomata to sugar, rind discs from green WT fruits were treated with artificial xylem sap solution (AXS, control), AXS supplemented with sorbitol (osmotic control), or AXS supplemented with 100 or 200 mM sucrose. Stomata that were treated with sucrose had significantly smaller apertures than those treated with AXS or the osmotic control (Figure 4). There was no significant difference between the stomatal closure of discs treated with 100 mM and the stomatal closure of discs treated with 200 mM sucrose. These results imply that the stomata on the fruit surface are functional and respond to known closing signals, similar to leaf stomata (Kelly et al., 2013).

### GCHXK Fruits Exhibited Lower Transpiration Rates Than WT Fruits

**FIGURE 5:**
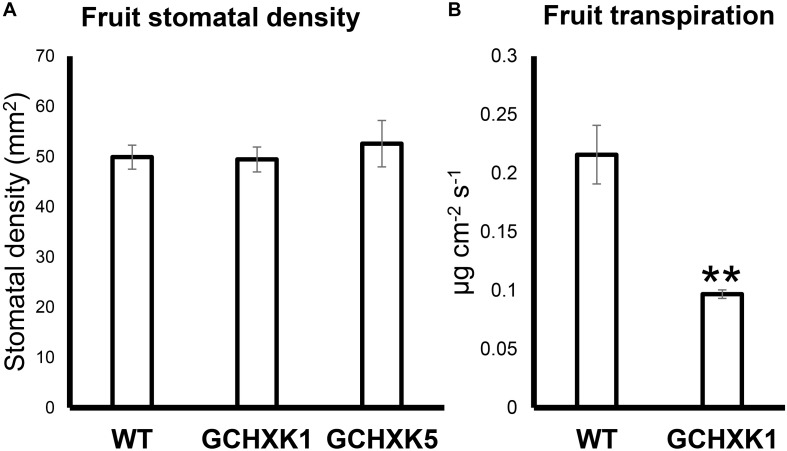
Expression of *AtHXK1* in guard cells of citrus fruits reduced fruit transpiration with no change in stomatal density. **(A)** Stomatal densities on WT, GCHXK1, and GCHXK5 fruits. For the WT, there were six biological repeats and for each GCHXK line, there were seven biological repeats. **(B)** Transpiration of green WT and GCHXK1 fruits, as measured with the LI-1600 steady-state porometer (WT: *n* = 8; GCHXK1: *n* = 12). Data displayed as means ± SE. Asterisks denote significant differences relative to the WT (*t*-test, *P* < 0.01).

It has previously been shown that sugar-sensing in guard cells is mediated by HXK and that expression of HXK in guard cells reduces leaf transpiration (Kelly et al., 2013; Lugassi et al., 2015). To examine whether HXK reduces the transpiration of fruit, we measured the stomatal density and transpiration of fruits from a previously described GCHXK citrus line (Lugassi et al., 2015). Stomatal density on 4-cm-diam., green GCHXK fruits was similar to that of WT fruits (Figure 5A). Yet, fruit transpiration of 4-cm-diam., green GCHXK fruits, measured using the LI-1600 steady-state porometer, was less than half of that observed for the WT fruits (Figure 5B).

**FIGURE 6:**
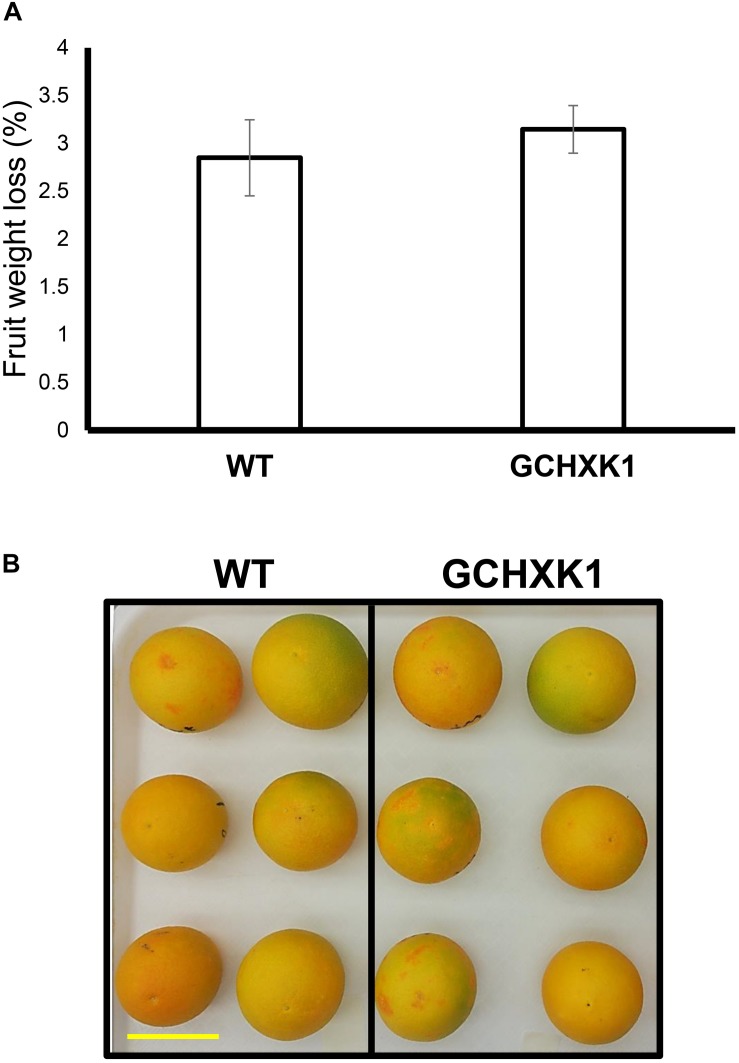
Water loss of ripe GCHXK fruits. **(A)** Ripe, harvested WT, and GCHXK1 fruits exhibited similar water loss after 5 days of long-day conditions (16 h light/8 h dark photoperiod) at 25°C (*n* = 6). **(B)** Ripe, harvested WT, and GCHXK1 fruits used for the water loss experiment, scale bar is 5 cm. Data displayed as means ± SE.

**FIGURE 7:**
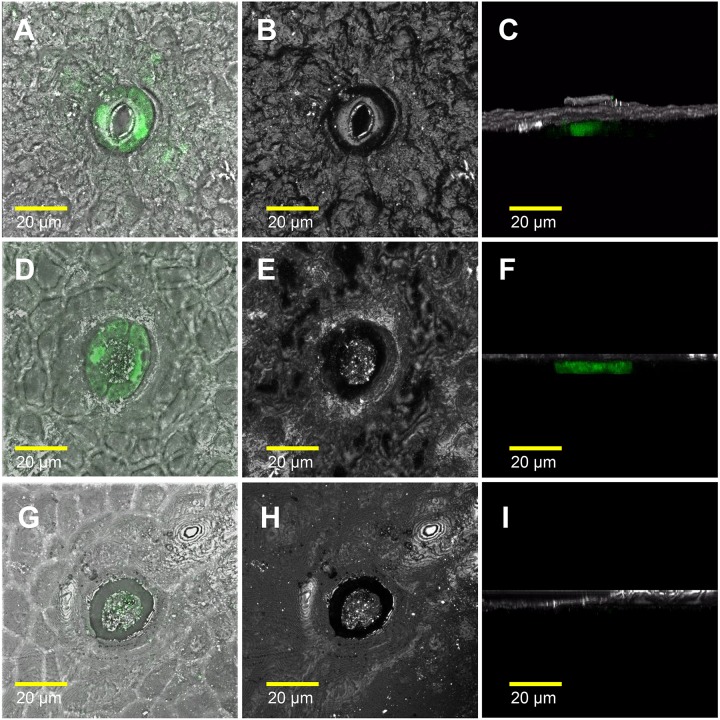
The stomatal pores of ripe yellow fruits are plugged. The confocal images **A**, **D**, and **G** are merged images of white light, reflections of the sample, and GFP fluorescence (stained green). The confocal images **B**, **E**, and **H** are reflections of the sample. The confocal images **C**, **F**, and **I** are 3D simulations, to provide a side view of the stomata, composed of reflections of the sample and GFP fluorescence (stained green). Breaker fruits were harvested from GCGFP plants. **(A–C)** Stomata were taken from a green segment of the fruit. **(D–F)** Plugged stomata from a yellow segment of the fruit; GFP staining can still be observed within the guard cells. **(G–I)** Plugged stomata from a yellow segment of the fruit in which GFP was not detected in the guard cells. Analyses were performed on three biological replicates.

We also measured water loss of ripened yellow fruits of similar size from GCHXK and WT plants following 5 days of exposure to long-day conditions (16 h light/8 h dark photoperiod) at 25°C (Figure 6). No difference in water loss was observed between the GCHXK and WT fruits (Figure 6). Confocal analysis of stomata of GCGFP citrus fruits revealed that, as demonstrated previously (Ben-Yehoshua et al., 1985; Hiratsuka et al., 2015), the stomatal pores of ripened yellow fruits are plugged (Figures 7D–I). However, GFP signal could still be observed in some of the plugged stomata (Figures 7D–F). These results suggest that green GCHXK fruits had lower transpiration rates, since the stomata at this stage are functional and respond to sugar. However, the functionality of the stomata disappears toward fruit ripening, since the stomata of yellow mature fruits are plugged and the plugged stomata do not respond to sugar signals (Figures 7A–C, compared to Figures 7D–I).

### GCHXK Seeds Are Smaller and Germinate More Slowly

**FIGURE 8:**
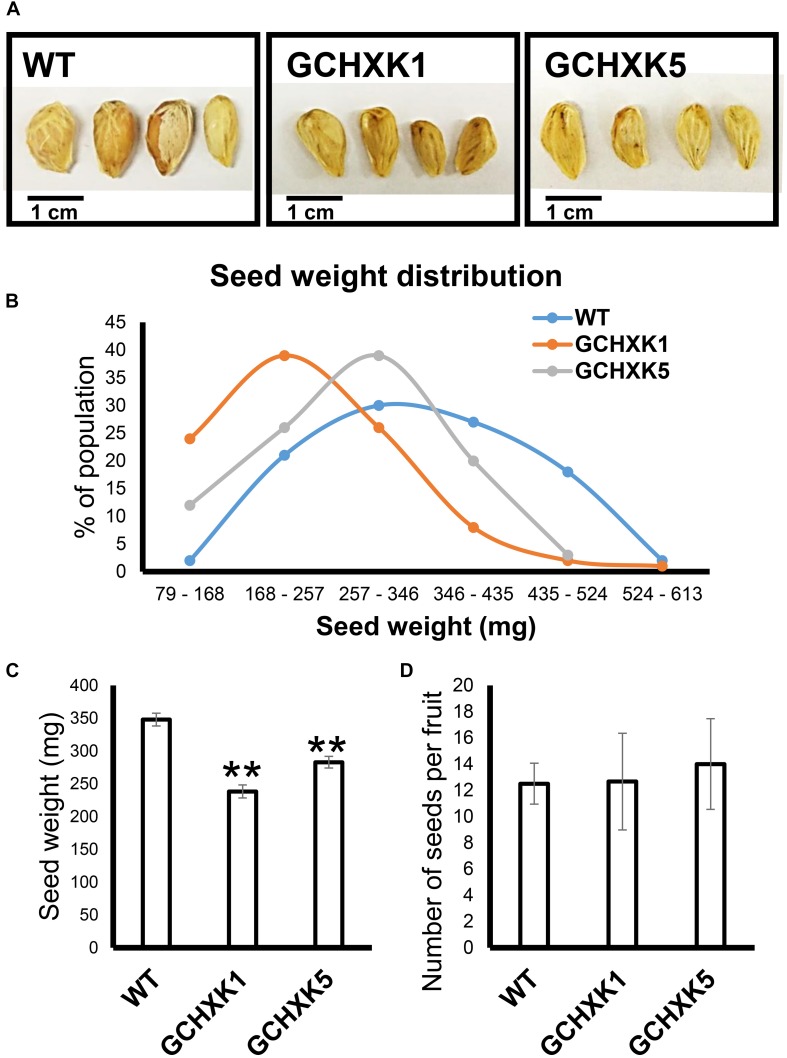
GCHXK citrus plants produce smaller seeds. Seed weight was determined by weighing 100 individual seeds from the GCHXK1, GCHXK5, and WT plants. **(A)** Representative images of WT, GCHXK1, and GCHXK5 seeds. **(B)** Weight distribution of the seeds. **(C)** Average weights of the WT, GCHXK1, and GCHXK5 seeds. **(D)** Number of seeds per fruit (for WT and GCHK1, *n* = 6 fruits; for GCHXK5, *n* = 3 fruits). Data are displayed as means ± SE. Asterisks denote significant differences relative to the WT (*t*-test, *P* < 0.01).

**FIGURE 9:**
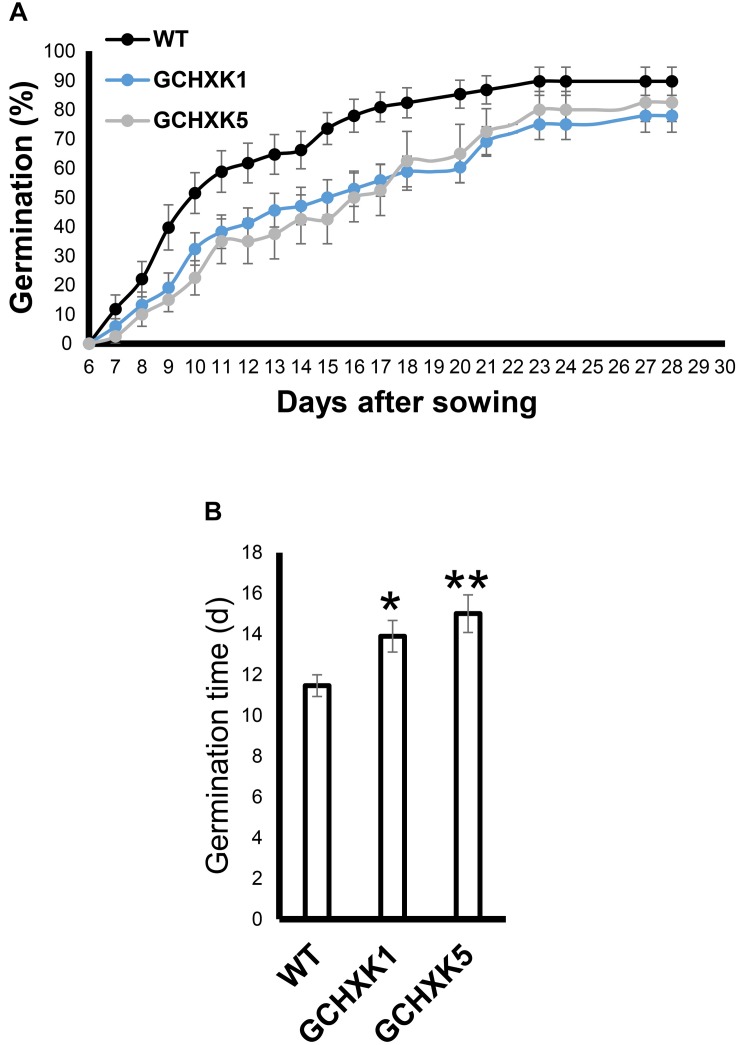
GCHXK seeds germinate more slowly than WT seeds. The germination rates of the different seeds were examined by sowing 68 seeds of the WT and GCHXK1, and 40 GCHXK5 seeds. The seeds were divided into groups of four, and the percentage of seeds that germinated was calculated from the average of the groups. **(A)** Percentage of seed that germinated over 28 days. **(B)** Average time to germination of WT, GCHXK1, and GCHXK5 seeds. Data are displayed as means ± SE. Asterisks denote significant differences relative to the WT (*t*-test, *P < 0.05; **P < 0.01).

Previous studies have suggested that the stomata of young citrus fruits allow photosynthesis and incorporation of CO_2_ by fruits (Hiratsuka et al., 2015), but the contribution of fruit photosynthesis to citrus fruit development is not known. We, therefore, took advantage of the isogenic background of WT and GCHXK lines to examine the potential effect of the lower stomatal conductance of GCHXK on fruit development. No significant changes were observed in size between the GCHXK and WT fruits, and the number of seeds per fruit of GCHXK plants was similar to that of WT plants (Figure 8D). Yet, the seeds of the GCHXK lines were significantly smaller, with a significant change in their weight distribution (Figures 8A–C). In addition, GCHXK seeds germinated significantly more slowly than WT seeds (Figure 9A). On average, the GCHXK seeds germinated 2–3 days later than the WT seeds (Figure 9B).

### Ripe GCHXK Fruits Do Not Show Reduction in Sugar Accumulation

**FIGURE 10:**
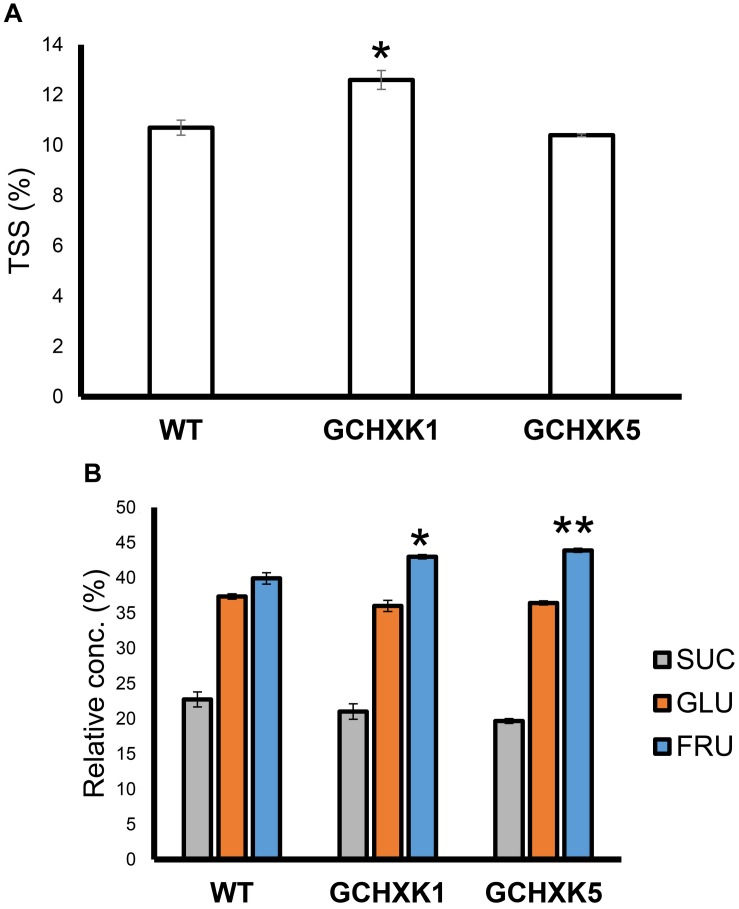
TSS content and sugar content of GCHXK fruit. **(A)** TSS levels in the juices of GCHXK lines, compared to the WT. **(B)** Relative sucrose, fructose, and glucose levels (from total sugar content) in the juice of GCHXK lines, compared to the WT. Each measurement included three samples. For the WT and GCHXK1, the juice of each sample was collected from two different fruits. For GCHXK5, the juice of each sample was collected from one fruit (due to a lack of fruits). Data are displayed as means ± SE. Asterisks denote significant differences relative to the WT (*t*-test, *P < 0.05; **P < 0.01).

To examine whether GCHXK affects the juice characteristics of mature yellow fruits, we analyzed the TSS and sugar contents of mature yellow fruits. The TSS content of the juice of one line, GCHXK1, was significantly higher than that of the WT, while that of GCHXK5 was similar to that of WT (Figure 10A). Sugar analyses revealed similar sucrose and glucose levels, along with fructose levels that were higher than those observed for the WT (Figure 10B). These results indicate that GCHXK has no negative effect on juice parameters.

## Discussion

In the current study, we used GCGFP plants that express GFP under the *KST1* promoter and found that stomata are formed on floral organs early in the reproductive phase. The *KSTpro:GFP* construct has already been proven to be very useful for monitoring the formation of guard cells (Kelly et al., 2017). It drives guard cell-specific expression soon after the differentiation of guard cells from guard mother cells is expressed in all guard cells and allows easy detection of the appearance of stomata and distribution (Kelly et al., 2017). In this study, stomata were observed on the sepals, styles, and anthers of closed citrus flowers, and on ovules upon the opening of the flowers. It is likely that the stomata on citrus anthers allow desiccation and the opening of the anthers, which is required for the release of pollen at anthesis, perhaps similar to the evolutionarily early role of stomata on the diploid sporophyte parts of mosses, which allow for spore desiccation and release (Sussmilch et al., 2017). Indeed, mutation of the *ICE1* transcription factor that reduces the number of mature stomata on Arabidopsis anthers has been shown to prevent anther dehiscence and the release of pollen (Wei et al., 2018). Yet, it has been suggested that stomata of closed flowers of *Lilium* hyb. *enchantment* anthers may allow assimilation of CO_2_ at very early stages of pollen development, by low photon intensity that might penetrate the closed flowers (Clement et al., 1997).

The role of stomata on the green parts of the flower (i.e. the sepals, ovule, and style) might be to allow CO_2_ uptake and photosynthesis (Vu et al., 1985). The functionality of fruit stomata has been demonstrated previously in several species such as banana (Johnson and Brun, 1966), purple passionfruit, yellow pitaya (Sánchez et al., 2013), and apple (Blanke and Lenz, 1989). It has also been shown that young green Satsuma mandarin (*C. unshiu*) fruits take up CO_2_ (Hiratsuka et al., 2012, 2015). Accordingly, the opening of fruit stomata by light (Blanke and Bower, 1991) and the closure of those stomata by sucrose support the notion that the stomata of young green fruits allow for photosynthesis. It has been reported that, at low light intensities, the photosynthesis of Satsuma mandarin fruit is more efficient than that carried out in its leaves (Hiratsuka et al., 2015). However, the extent to which fruit photosynthesis is important for fruit development is not known. Certain orange trees (Blanke and Bower, 1991) have small fruits, whose size has been partially attributed to inefficient fruit photosynthesis (Blanke and Bower, 1991). Another study examined why bagging of Satsuma mandarin (to prevent fungal, insect, and physical damage and to promote color development of the fruit skin) leads to reduced sugar levels at harvest. It was suggested that bagging probably inhibits photosynthesis and CO_2_ incorporation, leading to the lower sugar levels that were observed at harvest (Hiratsuka et al., 2012).

Yet, despite the reduced stomatal conductance, in our study, the sugar and TSS contents of mature GCHXK fruits were not lower than those of WT plants (Figure 10). However, the seeds of GCHXK plants were smaller and germinated more slowly, suggesting that the reduced stomatal apertures of GCHXK fruits did have a negative effect on seed development. Seed development starts at anthesis, immediately after pollination, when the fruits (ovules) are still very small, and since the stomata appear on ovules at anthesis, they may allow CO_2_ incorporation that contributes to ovule and seed development. Since we observed a reduction of >50% in GCHXK fruit transpiration, it is likely that the reduced apertures of the stomata of GCHXK fruits lead to lower fruit photosynthesis rates, which negatively affect seed development. No negative effects on leaf photosynthesis rates or plant growth were observed concurrent with the vegetative growth of GCHXK plants (Lugassi et al., 2015), minimizing the possibility that the seeds were indirectly affected by fluctuations in leaf photosynthesis.

As citrus fruits mature, the fruit guard cells collapse and the stomata accumulate a wax-like substance (Hiratsuka et al., 2015). Accordingly, no difference in water loss was observed between mature yellow GCHXK and WT fruits. Based on our GCGFP line, it appears that while the stomatal pore is plugged throughout fruit yellowing, some of the guard cells do not collapse, remain intact, and even retain their GFP signal (Figures 7D–F). Nevertheless, the results of this study indicate that the stomata on the reproductive organs at early developmental stages of citrus flowers are not only reminiscent of their epidermal origin, but may contribute to seed development.

## Data Availability Statement

All datasets generated for this study are included in the article/supplementary material.

## Author Contributions

NL, GK, AS, ER, NC, and DG planned and designed the research. NL and DG wrote the manuscript. NL, GK, TA, CF, YeY, and YoY performed the experiments. NL, GK, TA, YeY, ER, NC, and DG analyzed the data.

## Conflict of Interest

The authors declare that the research was conducted in the absence of any commercial or financial relationships that could be construed as a potential conflict of interest.
